# Myeloid dendritic cells are decreased in peripheral blood of Alzheimer’s disease patients in association with disease progression and severity of depressive symptoms

**DOI:** 10.1186/s12974-016-0483-0

**Published:** 2016-01-25

**Authors:** Antonio Ciaramella, Francesca Salani, Federica Bizzoni, Maria Donata Orfei, Carlo Caltagirone, Gianfranco Spalletta, Paola Bossù

**Affiliations:** Department of Clinical and Behavioral Neurology, IRCCS Santa Lucia Foundation, Experimental Neuro-psychobiology Lab, Via Ardeatina 306, 00179 Rome, Italy; Department of Neuroscience, University of Rome Tor Vergata, Via Montpellier 1, 00133 Rome, Italy; Neuropsychology Unit, University of Rome Tor Vergata, Via Montpellier 1, 00133 Rome, Italy; Menninger Department of Psychiatry and Behavioral Sciences, Baylor College of Medicine, Houston, TX USA

**Keywords:** Alzheimer’s disease, Mild cognitive impairment, Plasmacytoid dendritic cells, Myeloid dendritic cells

## Abstract

**Background:**

Dendritic cells (DCs) are major orchestrators of immune responses and inflammation. They are migratory cells, which may play a role in Alzheimer’s disease (AD), as suggested by prior in vitro studies. With the intent to investigate the clinical relevance of DC modifications in vivo, the present study was aimed to evaluate the levels of blood DCs in AD patients, in relation to the progression of the disease, the severity of its symptoms, and the treatment with acetylcholinesterase inhibitors (AChEIs), a class of drugs used to improve cognitive functioning in people with dementia.

**Methods:**

The two main subpopulations of immature blood DCs, namely myeloid (mDCs) and plasmacytoid (pDCs) cells, were evaluated by flow cytometry analysis in 106 AD patients, in comparison with the same cells from 65 individuals with mild cognitive impairment (MCI) and 73 healthy control subjects (HC). The relationship between blood DC levels and symptom severity was also assessed in AD patients, and their blood DC frequency was considered both in the absence or presence of treatment with AChEIs.

**Results:**

A significant depletion in blood mDCs was observed in AD patients, as compared to HC and MCI subjects. At variance, pDC levels were comparable among the three groups of subjects. The mDC decrease was evident only after the emergence of AD clinical symptoms, as confirmed by the follow-up analysis of a subgroup of MCI subjects who exhibited a significant decline in mDCs after their conversion to AD. Notably, the mDC decline was inversely correlated in AD patients with the frequency and severity of depressive symptoms. Eventually, the mDC depletion was not observable in patients treated with AChEIs.

**Conclusions:**

Our results provide the first evidence that blood mDC levels are dysregulated in AD. This phenomenon appears mainly linked to AD progression, associated with stronger severity of AD-related symptoms, and influenced by AChEI treatment. Taken all together, these data suggest that blood mDCs may serve as a cell source to test disease-induced and treatment-related changes and support the innovative notion that DCs play a role in AD, as ultimate evidence of the immune system participation in disease progression.

## Background

In association with amyloid plaques, reactive microglia have been broadly observed in AD brain. These cells are considered the resident mononuclear phagocytes, which act as the first line of brain defense, like macrophages in the periphery, and are associated with the release of many inflammatory factors in AD brains, such as complement factors, pro-inflammatory cytokines and chemokines [1]. Similarly, pro-inflammatory mediators (mainly cytokines and chemokines) are also increased outside the CNS of AD patients and in particular in their peripheral blood [2]. Peripheral immune changes seem to occur in AD patients also at cellular level, especially regarding the myelomonocytic cells. Even though blood monocyte percentage appears similar in AD and controls [3], in vitro-stimulated AD monocytes acquire a pro-inflammatory phenotype with secretion of higher levels of IL-6 [4]. Moreover, monocytes obtained from AD patients have a limited potential to differentiate into macrophages, which, in turn, show an impaired phagocytosis of amyloid β (Aβ) [5].

Following brain damage, an innate immune response takes place, mainly consisting of resident microglia and peripherally derived monocytes, macrophages [6], and, possibly, dendritic cells (DCs) [7]. Thus, it is not surprising that emerging data point to blood-derived mononuclear phagocytes as potential players in AD, as shown in mouse models [8–11]. DCs are the most potent professional antigen-presenting cells that play a central role in the initiation and modulation of innate and adaptive immune response [12], but their role in AD context is still largely unexplored. DCs may play an important role in brain diseases as they can reach CNS from periphery in both human diseases [13] and animal models [14] including AD [7]. Unfortunately, the characterization of DCs in human CNS is hard because of their relative paucity in the brain parenchyma, the in vivo analysis is difficult to perform, and the phenotypic characterization is complex, due to the absence of specific markers that are able to clearly distinguish DCs from microglia. Notably, before entering tissues and exerting their specific biological activity, immature DCs may circulate in the blood, so their analysis in the periphery would provide insights to better understand their involvement in brain diseases. More specifically, blood DCs represent less than 1 % of circulating mononuclear cells (PBMCs) in the peripheral blood, where they act as “immunological sentinels”. Two major subsets of human blood DCs, myeloid and plasmacytoid DCs (mDCs and pDCs, respectively), have been classified [15, 16]. Overall, both subsets of circulating DCs function as sentinels, which rapidly mature in response to antigenic stimulation, migrate to tissues, secrete cytokines, and effectively stimulate T cells. At variance with the pDC subset, mDCs are specifically considered precursor other than immature cells, since they fail to mature in response to TNF-α and express high levels of early myeloid markers, including CD33 [16]. Though the functional role of blood DCs and their subsets still needs to be clarified, a modification of their steady state has been often reported in ageing [17, 18] and in several pathological conditions, including CNS diseases and neurodegeneration [19–21], though not yet in AD. The knowledge about a potential involvement of DCs in AD is limited and mainly derived from in vitro data. We previously described that monocyte-derived DCs from AD patients show a more pronounced proinflammatory profile than DCs obtained from healthy control (HC) subjects [3]. This phenomenon could be associated to altered metabolism of Aβ, since monocyte-derived DCs obtained from HC and generated in vitro with Aβ_1–42_ peptide show functional alteration and increased production of inflammatory molecules [22]. In addition, we reported that monocyte-derived DCs from AD patients have a reduced ability to produce brain-derived neurotrophic factor following stimulation with Aβ_1–42_ peptide [23], providing evidence in support of the concept that myeloid DCs might participate in AD brain damage by promoting both inflammatory response and Aβ-dependent neurotoxic pathways.

Given the migratory nature of DCs and their potential recruitment to brain during neurodegeneration, the present study was addressed to confirm the possible participation of DCs in AD by evaluating the levels of the two DC subsets present in the blood of AD patients, in comparison with HC subjects. Then, in order to assess when blood DC changes occur during the progression of dementia, the blood DC analysis was performed also in a group of individuals with primary memory impairments, namely amnestic mild cognitive impairment (MCI), who are considered at increased risk to develop AD [24], as well as in a subgroup of MCI who converted to AD. With the intent to identify possible relationships between levels of blood DCs and clinical characteristics, the frequency of DC subpopulations in the blood was correlated with symptom severity in AD patients. Eventually, the impact on blood DC levels of acetylcholinesterase inhibitors (AChEIs), the drugs used as first line of symptomatic treatment of AD, was also taken into consideration.

## Methods

### Subjects

This study focused on 244 subjects consecutively recruited in the outpatient memory clinic of Santa Lucia Foundation in Rome, Italy. Among those, 106 patients were diagnosed with “probable AD”, 65 subjects with amnestic MCI, and 73 persons were included as HC. Medical and psychiatric history were obtained from each subject, and all patients underwent a series of standard clinical evaluations, including physical, neurological, and mental status examinations and brain magnetic resonance imaging. The study was approved by the Santa Lucia Foundation Ethical Committee, and, in accordance with the Helsinki Declaration, written informed consent was obtained from patients, patient representatives, or caregivers prior to enrollment.

Common exclusion criteria applied to all subjects were (i) major medical illness (e.g., cancer, obstructive pulmonary disease or asthma, hematologic disorders, active gastrointestinal, renal, hepatic, endocrine, cardiovascular diseases) and autoimmune inflammatory disorders (e.g., rheumatoid arthritis, type I diabetes, psoriasis, systemic lupus erythematosus, overt infections); (ii) comorbidity of primary psychiatric (i.e., schizophrenia, major depression onset before the AD onset) or neurological disorders (i.e., stroke, Parkinson’s disease (PD), seizure disorder, or head injury with loss of consciousness within the past year); (iii) known or suspected history of alcoholism or drug abuse; and (iv) computed tomography or magnetic resonance imaging evidence of focal parenchymal abnormalities.

Inclusion criteria for AD patients were (i) diagnostic evidence of probable AD consistent with the NINCDS-ADRDA criteria [25]; (ii) mild to moderate severity of dementia, defined as mini-mental state examination (MMSE) score ranging from 26 to 10 [26]; (iii) vision and hearing sufficient for compliance with testing procedures; and (iv) laboratory values within normal limits or considered not clinically relevant by the investigator.

Specific inclusion criteria for MCI were (i) diagnostic evidence of amnestic MCI consistent with Petersen guidelines [27]; (ii) a MMSE score ≥23. MCI patients were at the onset of their cognitive impairment and underwent their first clinical examination for the diagnosis of MCI. To better establish whether blood DC modifications are specific of AD and when they occur during disease progression, MCI patients were followed for two consecutive years at 6-month clinical follow-up visits aimed at verifying conversion to AD or to confirm the MCI condition. A subgroup of 11 MCI patients (16.9 % of the MCI cohort) who progressed over a 2-year time period to AD was eventually identified within the main group, indicated as MCI converters. They had blood drawn and DC analysis both at MCI baseline condition and at conversion.

The HC were neither related to one another nor to AD patients. Their inclusion criteria were (i) vision and hearing sufficient for compliance with testing procedures; (ii) laboratory values within the appropriate normal reference intervals; and (iii) neuropsychological domain scores above the cutoff scores, corrected for age and educational level, identifying normal cognitive level in the Italian population. Specific exclusion criteria for HC subjects were (i) dementia diagnosis, according with DSM-IV criteria or MCI according with Petersen criteria, and confirmed by the administration of the MDB and (ii) MMSE score <26 according with standardized norms for the Italian population [28].

### Demographic, cognitive, and neuropsychiatric assessment

Information on age, education, and sex was collected at baseline and verified by the patient’s informant. Global cognitive functioning was assessed with the MMSE [26], a commonly used neurocognitive screening test addressed to make the first dementia diagnosis and to assess its progression and severity. MMSE measures orientation, language, verbal memory, attention, visuospatial function, and mental control, with scores ranging from 30 (no impairment) to 0 (maximum impairment).

To evaluate the severity of participants’ neuropsychiatric symptoms, we administered to each participant the neuropsychiatric inventory (NPI) [29]. If appropriate, a family caregiver completed the NPI for the participant. The NPI was used to assess the frequency and severity of neuropsychiatric symptoms in 12 domains: delusions, hallucinations, agitation, depression, anxiety, euphoria, apathy, disinhibition, irritability, aberrant motor behavior, nighttime behavior disturbances, appetite, and eating abnormalities. Frequency was rated from 1 (occasionally) to 4 (very frequently) and severity from 1 (mild) to 3 (severe). If the symptom was absent, a score equal to 0 was given. The multiplication of frequency and severity was used as symptom composite score, with a range from 0 to 12.

To assess the patients’ ability to perform the activities of daily living independently, basic activities of daily living (ADL) were assessed using the Katz scale [30]. Instrumental ADL was assessed with Lawton and Brody’s I-ADL scale [31]. The ADL and the I-ADL scales range from severe functional impairment (higher scores) to fully functioning (lower scores). They were treated as continuous variables.

Among the 106 AD patients included in the study, 66 (62.3 %) were treated at baseline with the AChEI drug rivastigmine whereas 40 (37.7 %) were free from any AChEI treatment. Considering that taking AChEI may be a confounding factor for circulating immune cell analyses because of possible anti-inflammatory activity of these drugs, we split the AD group in the two subgroups of untreated (AD w/o AChEI) and AChEI-treated patients.

Furthermore, at the moment of the clinical assessment and blood sampling collection, the following fraction of group subjects were depressed and treated with antidepressants: 12.3 % of MCI and 10.3 % of AD patients. In particular, blood donors utilized the following antidepressants: paroxetine (1/65), sertraline (3/65), citalopram (1/65), and escitalopram (3/65) in MCI and paroxetine (1/106), sertraline (3/106), citalopram (1/106), and escitalopram (6/106) in AD.

Baseline characteristics of the HC, MCI, and AD subjects used in this study, including AD subgroups, were reported in Table 1. MCI subgroups, converters and no-converters, were described in Table 2. All groups were age and gender balanced according to chi-square test analysis (data not shown).

**Table 1:** Demographic and clinical characteristics of HC, MCI, and AD subjects

	HC	MCI	AD	AD w/o AChEI	AD AChEI
*n*	73	65	106	40	66
Gender (female, %)	57.2	57.0	51.9	55.0	50.0
Age (years)	71.5 ± 0.5	71.2 ± 0.7	72.1 ± 0.6	72.3 ± 1.0	71.9 ± 0.8
Education (years)	11.3 ± 0.5	9.0 ± 0.5*	8.5 ± 0.5**	9.0 ± 0.7*	8.1 ± 0.7**
MMSE	28.6 ± 0.1	27.0 ± 0.2**	20.9 ± 0.4**	22.0 ± 0.6**	20.0 ± 0.6**
ADL	5.9 ± 0.3	6.1 ± 0.05	8.5 ± 0.3**	8.0 ± 0.3**	8.9 ± 0.5**
IADL	6.7 ± 0.2	7.2 ± 0.2	13.8 ± 0.6**	12.9 ± 0.8**	14.6 ± 0.8**
NPI dep. (F × S)	1.3 ± 0.2	2.1 ± 0.2*	3.0 ± 0.2**	3.4 ± 0.4**	2.7 ± 0.3**

Data are expressed as mean ± SEM; *p < 0.005 and **p < 0.0005 vs HC

*MMSE* mini-mental status examination, *ADL* activities of daily living, *IADL* instrumental activities of daily living, *NPI dep. (F × S)* neuropsychiatric inventory–depression (frequency × severity)

**Table 2:** Demographic and clinical characteristics of MCI subjects at baseline

	MCI total group	MCI converters	MCI no-converters
*n*	65	11	54
Gender (female, %)	57.0	45.5	55.5
Age (years)	70.1 ± 0.7	72.4 ± 2.3	70.9 ± 0.7
Education (years)	8.8 ± 0.5	8.6 ± 1.2	9.1 ± 0.6
MMSE	27.1 ± 0.2	26.9 ± 0.7	26.9 ± 0.2
ADL	6.1 ± 0.04	6.6 ± 0.4	6.1 ± 0.04
IADL	6.9 ± 0.2	8.0 ± 0.8	7.1 ± 0.2
NPI dep. (F × S)	1.9 ± 0.2	0.7 ± 0.3*	2.4 ± 0.3
pDCs (%)	0.073 ± 0.005	0.077 ± 0.02	0.072 ± 0.005
mDCs (%)	0.26 ± 0.011	0.25 ± 0.02	0.26 ± 0.012

Data are expressed as mean ± SEM; *p < 0.05 vs MCI total group and MCI no converters

### Blood samples and flow cytometry analysis

**Fig. 1:**
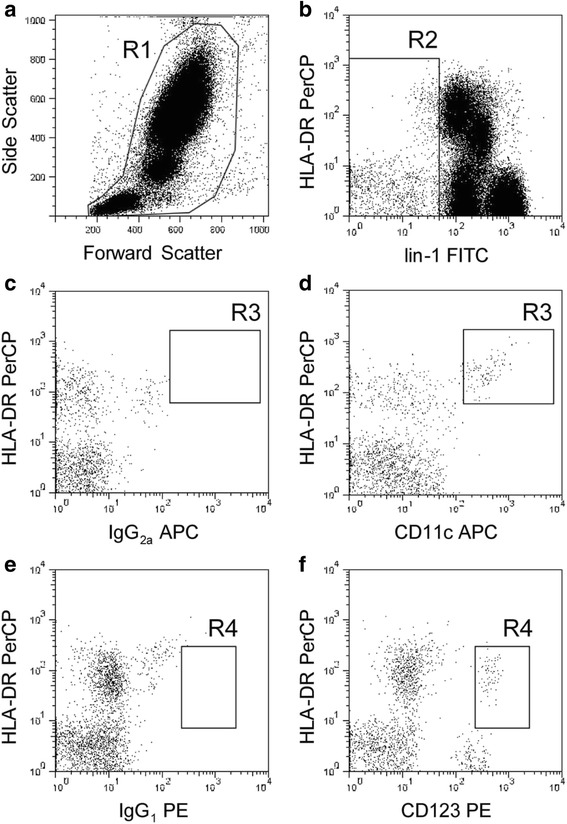
Representative dot plots showing the gating strategy to identify mDCs and pDCs by flow cytometry. Whole peripheral leukocytes were selected from live cells based on forward scatter and side scatter characteristics (**a**, gate R1). Blood DCs were then selected within R1 positive cells as negative for lineage markers (lin-1) (**b**, gate R2). Finally, blood DCs were identified as mDC (**d**, HLA-DR^+^/CD11c^+^/CD123^−^, gate R3) or pDC (**f**, HLA-DR^+^/CD11c^−^/CD123^+^, gate R4) in comparison to respective isotype control monoclonal antibodies (**c**, **e**)

Peripheral blood samples were obtained by venipuncture, collected into 10-ml tubes (EDTA Vacutainer, BD Biosciences, San Diego, CA, USA), processed, and stained within 4 h of collection. The blood draws were done in the morning to avoid the influence of circadian rhythm on levels of circulating immune cells. The frequency of peripheral blood DCs was assessed by flow cytometry analysis according to the manufacturer’s instructions and as described elsewhere [21]. In brief, 200 μL of whole blood were incubated for 20 min at room temperature with anti-lin-1 FITC (i.e., CD3, CD14, CD16, CD19, CD20, and CD56), anti-HLA-DR PerCP, anti-CD11c APC, anti-CD123 PE, and their isotype-matched monoclonal antibodies (all from Becton Dickinson, San Jose, CA, USA). Stained blood samples were subjected to erythrocytes lysis with 2 mL of FACS lysing solution (Becton Dickinson), vortexed and incubated for 10 min at RT in the dark. Following washing, samples were stored at +4 °C in the dark and analyzed within 1 h. Fifty thousand events were acquired with a four-color FACScalibur flow cytometer (BD Biosciences) running CellQuest software (BD Biosciences). Blood DCs were defined as lin-1^−^/HLA-DR^++^ cells. Blood DC subsets were identified using CD11c (mDCs) or CD123 (pDCs) antibodies. Representative panels that describe the gating strategy are reported in Fig. 1.

### Statistical analysis

Data are shown in dot plots and expressed as mean values ± SE. All statistical analyses were performed using Prism 4 software (GraphPad Software Inc). Comparisons between groups were made using the non-parametric two-tailed Mann-Whitney test for unpaired data or a two-tailed Wilcoxon signed rank test for paired data, as appropriate. Correlation analyses were made using Spearman’s rank test. Results were considered to be statistically significant when *p* value was < 0.05.

## Results

### mDC percentage is decreased in peripheral blood from AD patients as compared to MCI and HC subjects

**Fig. 2:**
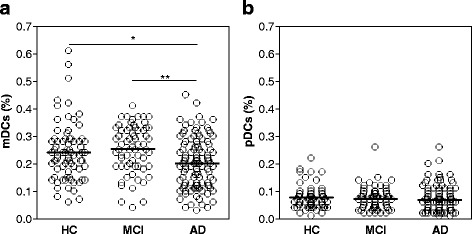
Peripheral blood mDCs decline in AD patients. The distribution of percentage values of mDCs (**a**) and pDCs (**b**) present in the blood of HC (*n* = 73), MCI (*n* = 65), and AD (*n* = 106) subjects is reported in the respective scatter dot plot, as indicated. The *black bars* indicate the mean. *p = 0.01, **p < 0.0001 (Mann-Whitney test)

We firstly investigated by flow cytometry the relative proportion of peripheral blood DC subsets, more specifically mDCs and pDCs, in the three main groups of subjects, namely HC, MCI, and AD. The clinical characteristics of the three groups of subjects are summarized in Table 1. As reported in Fig. 2a, the mean percentage of mDCs was significantly decreased in AD patients (0.20 ± 0.009), as compared to HC (0.24 ± 0.012, *p* = 0.01) or MCI (0.26 ± 0.011, *p* < 0.0001), while no statistically significant differences were observed between HC and MCI subjects. There were no differences in levels of pDCs among AD, MCI, and HC (Fig. 2b).

### Blood mDC reduction is observed after conversion from MCI to AD

**Fig. 3:**
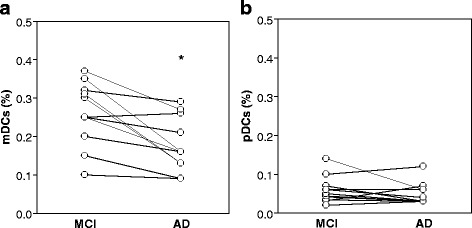
Peripheral blood mDCs decline with AD conversion. “Before and after” graph showing levels of mDCs (**a**) and pDCs (**b**) in a subgroup of 11 MCI converting to AD during a follow-up of 2 years. *p = 0.002 (Wilcoxon matched paired test)

With the aim to better investigate the blood DC decline during AD progression, we have taken into consideration a subgroup of MCI converters and compared their peripheral blood DC frequency between before and after conversion. The clinical characteristics of the MCI groups of subjects at baseline are summarized in Table 2. No main differences have been observed between the MCI converter subgroup and the whole MCI group as for age, education level, and gender distribution. As reported in Fig. 3a, most of the donors (9/11) showed a decrease in mDC levels following AD conversion. Overall, the mean percentage of mDCs was significantly decreased in MCI converter (0.25 ± 0.020) only after their conversion to AD (0.17 ± 0.021, *p* = 0.002). Furthermore, the levels of mDCs were similar comparing the total group of MCI, the subgroup of MCI which is not converted to AD and the subgroup of MCI that converts to AD analyzed before conversion (Table 2). No differences in pDC values were identified in MCI converters between before and after conversion (Fig. 3b).

### Relationship between mDC levels and depression in AD patients

**Fig. 4:**
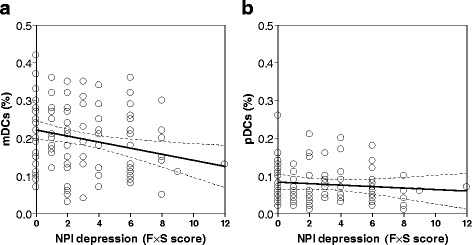
Relationship between depression symptoms scores and levels of mDCs in AD patients. The correlation between depression symptoms scores (NPI) and percentage values of either mDCs (**a**) or pDCs (**b**) present in the blood of AD patients (*n* = 106) is reported in the respective scatter dot plot, as indicated. The graphics show the tendency (*black lines*) and the 95 % confidence interval (*dotted lines*)

Correlation analysis was performed in order to assess possible associations between frequencies of blood DCs and functional assessment of AD patients, such as cognitive deterioration and behavioral and psychological symptoms. A significant inverse correlation was found between mDC levels and depression symptoms (*n* = 91, *p* = 0.01, rho = −0.25). As shown in Fig. 4, blood DC levels were lower in patients with higher NPI scores, as regards in particular the frequency × severity of depressive symptoms. Interestingly, this correlation was significant in the case of mDC subset (Fig. 4a), while not for pDCs (Fig. 4b). No significant correlations were identified in AD patients between blood DC subsets and the other 11 NPI subscales, ADL, IADL, or MMSE scores (data not shown). Finally, the treatment of AD patients with antidepressants did not appear to influence the above-described correlation. In fact, similar to the whole group of AD patients, a significant inverse correlation between mDC levels and NPI score of depression was observed in AD patients who did not undergo to any depression medication not treated with antidepressant drugs (data not shown). No associations between blood DC subsets and symptom severity were identified in MCI patients, both total group and MCI subgroups (data not shown).

### Blood mDC reduction is abated in AD patients treated with AChEI

**Fig. 5:**
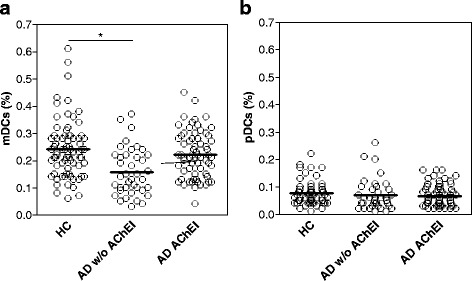
Treatment with AChEI modulates the level of mDCs in AD patients. The distribution of percentage values of mDCs (**a**) and pDCs (**b**) present in the blood of HC (*n* = 73), AD without AChEI treatment (*n* = 40), and AD treated with AChEI (*n* = 66) is reported in the respective scatter *dot plot*, as indicated. The *black bars* indicate the mean. *p < 0.0001

Since AD patients are commonly treated with cholinesterase inhibitors, which exert symptomatic effects and some delaying of disease progression, we ought to evaluate a potential interference of AChEI treatment on blood DCs frequency. Thus, we split our AD cohort into two subgroups, including AChEI-untreated and AChEI-treated patients and their characteristics are summarized in Table 1. As shown in Fig. 5a, the mDC mean percentage in untreated AD patients (AD w/o AChEI; 0.16 ± 0.014) was significantly decreased as compared to HC (0.24 ± 0.012, *p* < 0.0001), as well as AD patients receiving AChEI therapy (AD AChEI; 0.22 ± 0.011). At variance, the mDC levels in the AChEI-treated subgroup of AD were only slightly lower than those observed in HC, without any significant difference between them, suggesting that the anti-cholinesterase therapy may mitigate the blood mDC reduction observed in AD patients. No differences among the two groups of AChEI-untreated and AChEI-treated patients and HC were observed regarding the levels of blood pDCs (Fig. 5b).

## Discussion

In recent years, DCs have emerged as a promising research area to investigate CNS diseases’ pathophysiology and although a number of studies showed that DCs play critical roles in CNS inflammation, particularly during stroke or multiple sclerosis (MS) [20, 32], less evidence points to their potential role in neurodegenerative diseases. In this context, our data support the hypothesis that blood DCs are altered in AD patients.

Our main finding is that AD patients have decreased levels of the myeloid subset of blood DCs, as compared to matched HC. DC reduction is probably a general phenomenon in several full-blown diseases, since it has been featured in autoimmune diseases [33–35], infections [36], cancer [37], and CNS disturbances [19–21]. The blood DC reduction can be due to alterations in DC viability, DC mobilization, or their impaired differentiation from progenitors. Hence, the fate of DCs missing from the bloodstream is not always the same. In some cases, blood DCs specifically migrate from the blood to the diseased tissue. This situation was described in stroke or MS, where DCs have been found in the brain [20, 38], or in coronary artery disease, where DCs have been identified in atherosclerotic plaques [39]. On the contrary, DCs may die following infection, as in malaria [40], HIV infection [41], or severe sepsis [42], where infected DCs have been shown to undergo apoptosis. This latter circumstance seems unlikely to happen in AD patients, where we did not observe an abnormal number of apoptotic DC in the peripheral blood (data not shown). Conversely, as previously demonstrated in stroke and MS, we speculate that in AD, a significant percentage of blood mDCs move from peripheral blood probably reaching the brain of patients, maybe at choroid plexus or meninges level, where DCs could sample cerebrospinal fluid content. To this regard, a part of Aβ peptide efflux has been described to occur in AD exactly from these CNS elimination routes [43]. After DCs have reached these privileged positions, and sampled brain antigens, they may respond to neuroinflammation signals and react to neurodegeneration [44]. Importantly, the recent and accurate description of a CNS lymphatic system connected to deep cervical lymph nodes, which are able to drain cerebrospinal fluid and carry immune cells such as CD11c^+^ [45], leads to more thorough investigation of the leukocyte presence in CNS immune responses in neurodegeneration and supports our hypothesis about blood-derived DC involvement in AD brain pathology.

Notably, we observed a specific reduction of myeloid DCs, while pDC subset appears mostly unchanged. This is in line with recent GWAS studies where the involvement of numerous immune-related genes as risk factors for AD was highlighted. Among these genes, there are CD33 and TREM2 notoriously associated with myeloid cell function. These studies strongly suggest that myeloid compartment of the immune system is a crucial component of susceptibility to AD [46]. Although it is still unclear to what extent peripheral myeloid cells engraft in the AD brain, it has been observed that CD33 expression levels are increased in CNS, but appeared decreased in peripheral mononuclear cells of AD patients [47], suggesting that central and peripheral myeloid cell frequency may be linked processes during the disease. Furthermore, the specific implication of the mDC subset in AD is consistent with a more pronounced pro-inflammatory feature of this subpopulation as compared to pDCs [48], also converging with our previous in vitro studies, which showed that monocyte-derived DCs acquire a pro-inflammatory profile in AD conditions [3, 22, 23].

Differently from AD, we recently reported that in PD patients, there is a reduction of blood DCs both in mDCs and pDCs [21]. This last observation suggests that mDC decrease in the blood may be a common phenomenon in patients with neurodegenerative diseases, though DC subsets may be endowed with different functions in neuroinflammatory pathway linked to neurodegeneration evolution, depending on the type of specific neurological disturbance.

In the present study, we propose that blood mDC decrease appears specifically linked to AD progression, since the immune cell perturbation becomes evident in MCI subjects only after their conversion to AD while it is not evident at MCI level. Further, the blood mDC decrease is associated with severity and frequency of the depressive episodes and comes out mitigated in patients treated with the AChEI drugs used to slow down disease progression.

In fact, regarding the MCI subjects, namely individuals at increased risk to develop dementia, we observed that mDC levels were not different from those observed in control donors. This was true not only for the whole group of MCI but also for MCI converters, the retrospectively identified subgroup of MCI patients who have subsequently converted to AD, hence in the earliest phase of AD. This indicates that blood mDC depletion specifically occurs only after the onset of AD symptoms suggesting that DCs may be involved in AD progression, but not in the earlier phases of dementia.

We also found low mDC levels associated with increased depressive severity suggesting that depletion of mDC subset in AD may be a functional feature of neuroinflammation-dependent neurodegenerative symptoms. The possibility that DC alteration may be linked to depressive symptoms is not very surprising, since depression and dementia share many common pathophysiological features with chronic inflammation, activation of the hypothalamic–pituitary–adrenal axis and deficit of neurotrophin signalling [49].

Finally, we observed that reduction of blood mDCs did not occur in AD patients treated with AChEI. These drugs, recommended as first-line treatment option for patients with mild to moderate AD, exert symptomatic effects and delay the natural course of the disease, supporting the hypothesis of an association between blood mDC perturbation and AD progression. Interestingly, AChEI treatment has been also reported to have beneficial effects on behavioural disturbances, including depression [50], further strengthening the suggested link between mDC frequency, AD progression, and depressive symptoms. In addition, AChEI, which are commonly used to compensate for acetylcholine depletion in AD brains, other than enhancing cholinergic transmission, may also exert anti-inflammatory activity on both neuroinflammation [51] and systemic inflammation [52]. Since a recent study showed that acetylcholine modulates functions of DCs cultured in vitro through an autocrine/paracrine loop [53], further studies are highly recommended in order to identify the anti-inflammatory mechanisms of AChEI, DC-mediated.

### Limits of the study

To our knowledge, this is the first study that analyzes the main subpopulations of circulating DCs in AD patients. Below are some points listed that should be investigated further. (i) We hypothesize that the main remark of this study, namely the reduction of mDCs from blood of AD patients, is associated with the recruitment of these cells in the brain. However, we did not investigate directly the fate of DCs, but we supposed their recruitment in the brain, taking in consideration previously reported studies [20, 38]. Although clarifying the fate of myeloid DCs would allow to describe the phenotypic and functional features of these cells missing from the blood of AD patients, it is hindered by the difficulty to monitor the patients’ brain for infiltrating myeloid cell subpopulations. (ii) We performed a cell staining strategy that allowed us to identify two different subpopulations of circulating DCs (lin-1^-^/HLA-DR^++^/CD11c^+^/CD123^-^, myeloid DCs and lin-1^-^/HLA-DR^++^/CD11c^-^/CD123^+^, plasmacytoid DCs) but a more detailed DC characterization by BDCA’s analysis [54] may prove useful to identify further distinctive DC subsets involved in AD. (iii) Finally, this study did not take into account the analysis of AD biomarkers and neuroimaging of brain inflammation, whose possible correlation with blood DC levels might add to our findings. The above limits are open questions which deserve to be clarified in future studies.

#### Blood DCs as potential biomarkers

In recent years, it has been shown that DCs may be a source of molecular marker for many diseases. For example, it has been shown that in lung tumors, mature DCs represent a specific marker for tumor-associated local lymph node-like structures [55] and, in some forms of leukemia, a specific subpopulation of BAD-LAMP^+^ pDCs identifies transformed pDCs [56]. In diabetes, it has been suggested that pDCs could predict Th1-mediated type 1 disease [57] while, in systemic lupus erythematosus (SLE), there is strong genetic evidence that pDCs are critically involved in pathogenesis and autoantibody production [58]. The concept of immune processes as AD biomarker resource in neurodegenerative diseases is quite at the beginning. In particular, it has been shown that AD-derived monocytes exhibit an impaired differentiation into macrophages in vitro and also a limited phagocytic ability with a surface uptake of Aβ undergoing apoptosis after Aβ exposure [5]. Interestingly, a decreased abundance of monocytes was described in MCI compared with AD patients, suggesting a transient decline in the number of monocytes in the early stages of the disease [59]. More recently, lower levels of absolute percent of CD14 cells in early AD patients and an altered expression of HLA-DR, CD16, and CCR2 were found [60]. All studies reported above, including our findings, suggest that myeloid-derived cells, starting from monocytes to DCs, are impaired in AD, leading to ask what is the real contribution of these cells to the development of AD and whether they may be considered for further development of clinically relevant tools.

## Conclusions

We identified blood mDCs as a cell population of high potential interest in AD progression and treatment, even though future longitudinal studies accompanied by evaluation of biomarkers, like those searching for Aβ and tau proteins in cerebrospinal fluid, and specialized PET and MRI scans may be useful to better understand the meaning of mDC decline in AD clinical course.

In conclusion, this study provides the first evidence that blood DCs, specifically those of myeloid origin, are dysregulated in the blood of AD patients, fostering the concept that blood immune cells may play a key role in inflammatory-mediated progression of AD.
